# Kinetic Study of the Biodegradation of Acephate by Indigenous Soil Bacterial Isolates in the Presence of Humic Acid and Metal Ions

**DOI:** 10.3390/biom10030433

**Published:** 2020-03-11

**Authors:** Simranjeet Singh, Vijay Kumar, Sourav Singla, Minaxi Sharma, Dhananjaya P. Singh, Ram Prasad, Vijay Kumar Thakur, Joginder Singh

**Affiliations:** 1 Department of Biotechnology, Lovely Professional University, Phagwara 144411, Punjab, India; simnav14@gmail.com (S.S.); souravsingla2011@gmail.com (S.S.); 2 Regional Ayurveda Research Institute for Drug Development, Gwalior 474009, MP, India; vkumar8491@gmail.com; 3 Department of Food Technology, Akal College of Agriculture, Eternal University, Baru Sahib 173101, HP, India; minaxi86sharma@gmail.com; 4 Crop Improvement, Division ICAR- Indian Institute of Vegetable Research, Jakhini (Shahanshapur), VARANASI 221 305, UTTAR PRADESH, India; dhananjaya.Singh@icar.gov.in; 5 Department of Botany, Mahatma Gandhi Central University, Motihari 845401, Bihar, India; 6 Enhanced Composites and Structures Center, School of Aerospace, Transport and Manufacturing, Cranfield University, Bedfordshire MK43 0AL, UK

**Keywords:** acephate, humic acid, metal stress, *Pseudomonas*, toxicity

## Abstract

Many bacteria have the potential to use specific pesticides as a source of carbon, phosphorous, nitrogen and sulphur. Acephate degradation by microbes is considered to be a safe and effective method. The overall aim of the present study was to identify acephate biodegrading microorganisms and to investigate the degradation rates of acephate under the stress of humic acid and most common metal ions Fe(III) and copper Cu(II). *Pseudomonas azotoformans*s strain ACP1, *Pseudomonas aeruginosa* strain ACP2, and *Pseudomonas putida* ACP3 were isolated from acephate contaminated soils. Acephate of concentration 100 ppm was incubated with separate strain inoculums and periodic samples were drawn for UV—visible, FTIR (Fourier-transform infrared spectroscopy) and MS (Mass Spectrometry) analysis. Methamidophos, S-methyl O-hydrogen phosphorothioamidate, phosphenothioic S-acid, and phosphenamide were the major metabolites formed during the degradation of acephate. The rate of degradation was applied using pseudo-first-order kinetics to calculate the half-life (t_1/2_) values, which were 14.33–16.72 d^−1^ (strain(s) + acephate), 18.81–21.50 d^−1^ (strain(s) + acephate + Cu(II)), 20.06 –23.15 d^−1^ (strain(s) + acephate + Fe(II)), and 15.05–17.70 d^−1^ (strains + acephate + HA). The biodegradation efficiency of the three bacterial strains can be ordered as *P. aeruginosa* > *P. putida* > *P. azotoformans*. The present study illustrated the decomposition mechanism of acephate under different conditions, and the same may be applied to the removal of other xenobiotic compounds.

## 1. Introduction

In the current scenario, the use of pesticides is obligatory to save crops from various adversaries such as pests, insects, fungi, bacteria, weeds, etc. In India, pests are responsible for destroying crops worth an economic value of 46 billion US dollar [1]. The Green Revolution caused a steep increase in the use of pesticides in India. Presently, the annual consumption of pesticides in India is 58000 tones, i.e., 270 g/ha. The crop-wise consumption of pesticides is distributed in the following order: cotton (66.71%) > arhar (64.75%) > jute (53.27%) > paddy (48.12%) > maize (25.11%) [2]. It is assumed that the applications of pesticides may increase in the future to nourish the fast mounting population as the land is increasingly used for agricultural activities [3,4].

Pesticides, including their metabolites, are harmful to every aspect of the environment (i.e., soil, water, and air). Pesticides storage and applications lead to the production of harmful metabolites, which can pollute air, water, and soils through horizontal and vertical movements [5]. The remediation of pesticide-contaminated land and sites has relied on conventional methods including pyrolysis, incineration, chemical decomposition, spillage into sewage, and landfill [6]. These are costly, time-consuming, and inefficient techniques and can lead to the formation of toxic metabolites, i.e., side-products of pesticides. On the other side, the use of microorganisms is an effective way to decompose pesticides in a green or eco-friendly manner [7]. These microbe-based techniques are efficient in eradicating hazardous compounds from different ecosystems. Many microorganisms have been isolated from soils around the world, which can decompose a wide variety of pesticides.

Acephate (N-(Methoxy-methyl sulfanyl phosphoryl)-acetamide) is an organophosphate pesticide (OP). It has been widely used for agricultural and public health purposes and is one of the most common and cheapest insecticides in India. It has been widely applied in plant protection, timber protection, sugar and maize plantations, termite control, and vector control [8]. This insecticide is registered under more than 10 trade names, and annual imports exceed 1000 tons. Acephate and its metabolites are highly soluble in water, and their leaching to soil and water is an important issue. The literature revealed that acephate and its metabolites, including methamidophos, have been listed among the major contributors to soil and water pollution [9]. To overcome this problem, various authors have isolated numerous microbes decomposing these insecticides (acephate and its metabolite methamidophos) in a green way. Numerous studies have been reported on the biodegradation of methamidophos, and only three studies were found on the biodegradation of acephate [7,9,10], which report that isolated microbes can decompose acephate and other organophosphate pesticides under different environmental conditions.

The bio-degradation rate depends on various parameters and can be enhanced by different activators such as humic substances and several activated metal ions [7]. Activators like humic acid significantly reduce the toxicity and encourage microbes to decompose pesticides without any hindrance. Active metal ions can decompose the OPs through bond cleavage mechanisms [11]. A combination of microbes with an optimized dose of active metal ions may enhance the decomposition rate. On the basis of our previous results, and to promote an environment-friendly approach, this research aimed to examine the biodegradation potential of some *Pseudomonas* strains against acephate and the influence of humic acid and metal ions [Fe(III) and Cu(II)] on biodegradation efficiency [7]. The major reason behind the selection of Fe(III) and Cu(II) was the multiple applications of both in living systems. Cu(II) and Fe(III) are very important micronutrients of soil and can improve plant health through a complex mechanism. Inadequate distributions of these ions may alter the crop production as well as human health.

## 2. Materials and Methods

### 2.1. Chemicals and Reagents

Technical grade acephate (purity > 98%) was obtained from Gautmi Pvt. Ltd., Hyderabad, India. M9 medium was composed of, KH_2_PO_4_ (3 g/L), NH_4_Cl_2_ (1 g/L), Na_2_HPO_4_ (6 g/L), and NaCl (0.5 g/L), at pH (7.2 ± 0.2); 1 M sterilized CaCl_2_ (0.1 mL) and MgSO_4_ (2 mL) were also added to the medium. Ferric chloride and humic acid were purchased from Loba Chemie (Mumbai, India) and M9 media from Himedia (Mumbai, India). All the chemicals were of the highest AR grade.

### 2.2. Soil Sample Collection and Isolation of Microorganisms

Soil samples were collected from industrial soil in Jalandhar, district of Punjab, India (latitude 31.32560 E and East longitude 75.57920 E, at an elevation level of 228 m above the sea level). A soil auger was used to collect the soils samples, and 1 kg of soil sample was collected.

Soil samples were sieved using a 2-mm pore-sized sieve and thoroughly homogenized using a pestle and mortar. Bacterial isolates were obtained by the enrichment culture technique; particular microbes were isolated on the basis of their acephate usage capability. About 5 g soil sample was inoculated in a 50-mL Erlenmeyer flask containing M9 media supplemented with acephate at 100 mg/L concentration as a sole source of carbon, nitrogen and phosphorous. Cultures were incubated at 30 °C for 7 days on a rotatory shaker (150 rpm) under dark conditions. After 7 days, 1 mL culture suspension was inoculated to fresh M9 medium containing acephate (100 mg/L) and incubated also for 7 days. The cultures were acclimatized 5 times with acephate and thereafter sub-cultured in the presence of acephate containing agar, and bacterial colonies appeared on the Petri plates. Single colonies were selected and mass multiplied in broth M9 media (without agar) for degradation studies. The isolated strains were further identified on the basis of biochemical, morphological, and molecular analysis.

### 2.3. Molecular Characterization of Bacterial Isolates

**Figure 1:**
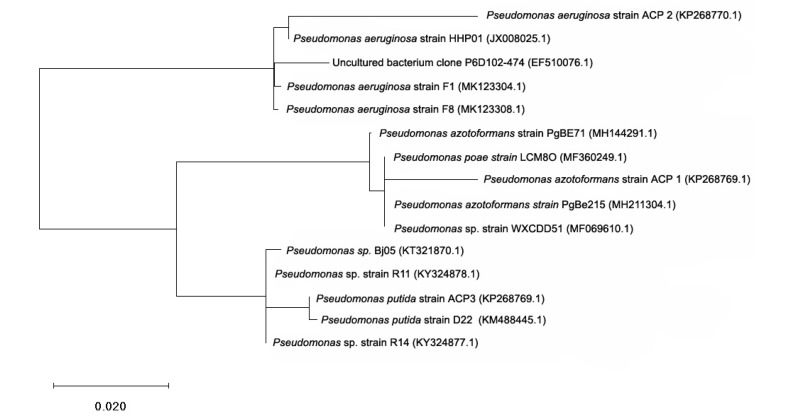
Phylogenetic tree of isolated *Pseudomonas* strains from Jalandhar, Punjab, showing relations of the strains ACP1, ACP2, and ACP3 with the closest relatives on the basis of 16S ribosomal RNA gene sequence analysis.

16 S ribosomal RNA (rRNA) gene analyses of the individual bacterial isolates were conducted at Samved Biotech Pvt. Ltd. (Ahmedabad, India). To authenticate the identity of all the four isolates, gene fragmentation of 1.5 kb 16 S rRNA was amplified using the total DNA of each isolate, and later on, sequenced using universal primers 1492R (ACCTTGTTACGACTT) and 27F (AGAGTTTGATCMTGGCTCAG). The DNA extraction from bacterial isolates and amplification using PCR and phylogenetic analysis were performed [1,4] and are well defined in the Figure 1.

### 2.4. UV—Visible Spectrophotometric Study for Cell Growth

To determine the growth rate of isolates, the concentration-dependent degradation of acephate was performed at regular intervals, where each flask was forfeited for different conditions and concentration. Monitoring of cell growth was done using a UV—visible spectrophotometer (Shimadzu-1800 Schweiz GmbH, Germany) at 600 nm, along with distilled water as blank. The percentage growth and optical density (OD) was calculated using the following formula: Percent growth = [(A − A_0_)/A] × 100; where A_0_ = initial OD and A = OD with time (3rd to 30th day).

### 2.5. Biodegradation of Acephate

To perform the degradation experiments, strains were pre-cultured in M9 using 100 ppm concentrations of acephate. Flasks were incubated on a dark orbital shaker at 120 rpm overnight at 30 °C. The content of the inoculated flasks, including M9 medium and acephate, was centrifuged and analyzed. To determine the effect of Cu (II), Fe (III), and humic acid, the same experiment was repeated under the same conditions, respectively. Flasks without M9 medium having a concentration of 100 ppm acephate served as controls. All the experiments were conducted in triplicate.

### 2.6. Characterization of Degradation Metabolites of Acephate by Using ESI-MS and FTIR Analysis

Liquid chromatogram, coupled with mass spectrometry (electrospray ionization ESI-MS) was used to characterize the degradation metabolites of acephate. To collect the samples from various experiments, we followed the methodology described in our recent report [1]. In brief, 100 mL of the spent medium was clarified by centrifugation at 5000 rpm, followed by filtration through a Whatman 1 filter paper. The clarified medium was extracted thrice with an equal volume of ethyl acetate. The extracted organic phase was allowed to air dry, and the remaining residue was dissolved in a minimal volume (250 mL) of water, and about 1 µL was taken for mass analysis using a mass spectrophotometer (Waters, Q-TOF Micromass, Manchester, UK). Functional groups of acephate and its decomposed metabolites were identified by applying FTIR analysis. FTIR analysis was performed on a Shimadzu-8400s FTIR spectrophotometer (Shimadzu, Japan) using KBr pellets of the dry mass of technical grade acephate and extracted or decomposed metabolites. The FTIR analysis was performed in the mid-IR region of 400–4000 cm^−1^ with a 16 scan speed [10,11,12].

### 2.7. Degradation Kinetics

The decomposition rates of the acephate under different compositions were analyzed according to pseudo-first-order kinetics, as mentioned in our recent report [1,3]. Briefly, Equations (1)–(3) correspond to the equations of the concentration variation with time plotted on the basis of the linear regression results obtained by plotting time (in the day) vs. Log C_t_ (in ppm).

d[C]/dt = −k_obs_[C] (1)

log[C]/[C]_0_ = k_obst_ (2)

t_1/2_= (1/k_obs_) × log 2 (3)

where, [C] = acephate concentration at time t (ppm), [C]_0_ = initial acephate concentration (ppm), k_obs_ = pseudo-first-order constant (day^−1^), which is equal to slope of line (i.e., k_obs_ = −slope) [13]

### 2.8. Statistical Analysis

Experimental data were analyzed using MS Excel for the calculation of means and standard errors. Analysis of variance (ANOVA) was carried out using SPSS 16 statistical software (Chicago, Illinois).

## 3. Results

### 3.1. Isolation and Identification of Acephate Degrading Bacterial Strain

Isolated strains were characterized using various biochemical tests, namely MR test, catalase test, citrate test, indole test, gram staining test, and 16S rRNA analysis (Figure S1 and Table S2). 16S rRNA sequence analysis was used to identify the bacterial isolates. Homology search using BLAST revealed congruence of the three sequences with 16S rRNA sequences of *Pseudomonas* family (*P. azotoformans* ACP1, *P. aeruginosa* ACP2, and *P. putida* ACP3). A phylogenetic tree constructed using Mole-Blast showed a close relationship of the three bacterial isolates, and the sequences were deposited in GeneBank under accession numbers KP268769.1, KP268770.1, and KP268771.1, respectively (Figure 1).

### 3.2. Cell Growth Studies Using UV—Visible Spectrophotometric Technique

The tolerance of acephate, humic acid, and metal ions to bacterial strains was tested using the minimum inhibitory concentration (MIC) study. The optimized concentration level of acephate, humic acid, Fe(III), and Cu(II) was 100, 10, 25, and 15 ppm, respectively. Cell growth of individual bacterial strains was checked at 600 nm (OD = 600) using UV—visible spectroscopy. An increase in optical density (OD) at 600 nm has demonstrated the consumption of acephate as a source of carbon and phosphorus [7]. Cell growth represents the decomposition, which was time-dependent and inversely proportional to the concentration of acephate, humic acid, and metal ions (Fe and Cu) (Table 1). The results indicated that all three bacterial isolates were able to degrade acephate in M9 medium in the following order: *P. aeruginosa* > *P. putida* > *P. azotoformans*. With the application of acephate (100 ppm) and inoculums of three bacterial strains in separate experiments, more than 68% degradation of acephate was observed within 30 days. The observed OD growth was between 6 –79% in the order of *P. aeruginosa* > *P. putida* > *P. azotoformans*. The values of OD (at 600 nm) increased from 0.11 ± 0.025 to 0.19 ± 0.032 within 30 days. A similar order was noticed when experiments were performed in the presence of humic acid (68–75%). In the case of humic acid, initial cell growth was slow, but increased after the 7th day and almost reached the level of the experiments without humic acids. With the addition of metal ions, i.e., Fe and Cu, the initial values of OD increased, but after 30 days, the percentage OD decreased (Table 1). The decrease in OD with the inclusion of metal ions may be due to the toxicity of metal ions at a higher concentration level [7]. In the presence of metal ions, the OD growth was 63–69% for Cu(II) and 60–67% for Fe(III) (Table 1). The observed order of cell growth was similar to that mentioned above, i.e., *P. aeruginosa* > *P. putida* > *P. azotoformans*. It was noticed that metal ions adversely affected cell growth relative to the experiments without metal ions.

### 3.3. Biodegradation Products Identification and Confirmation by Using MS and FTIR Studies

**Figure 2:**
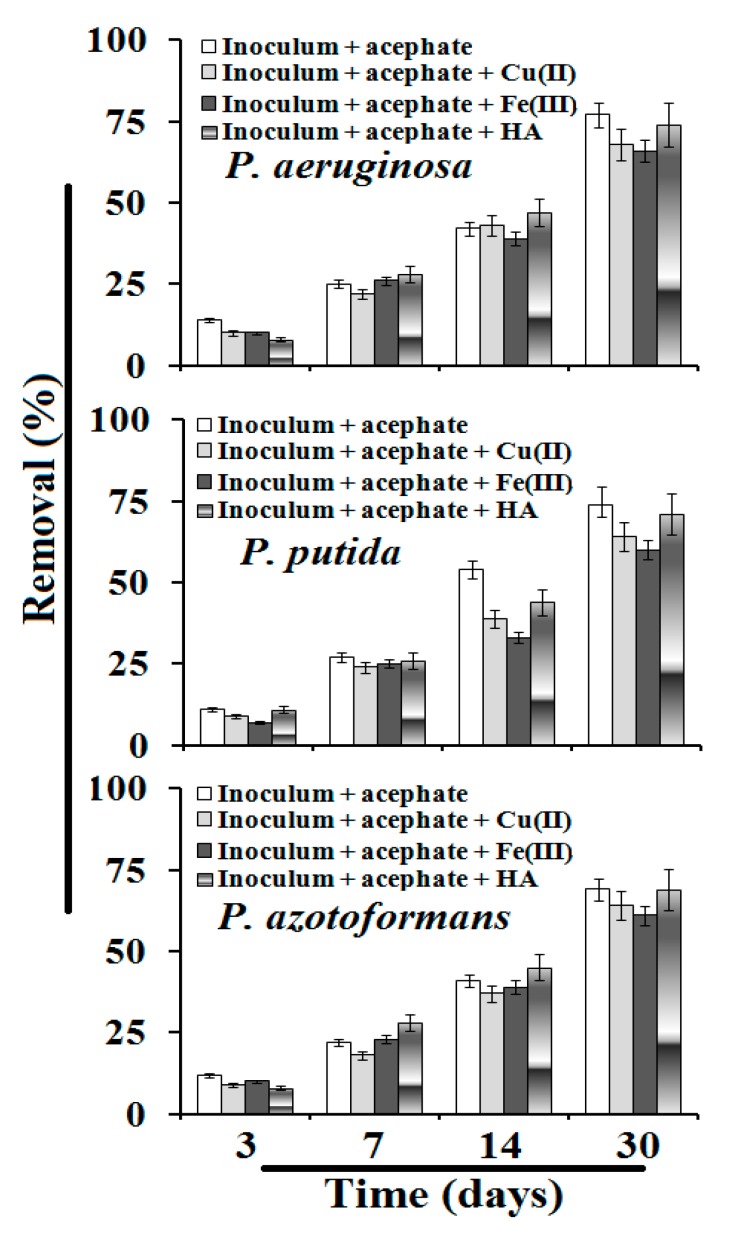
Removal (%) of acephate after incubation with three strains in M9 medium under different conditions.

**Figure 3:**
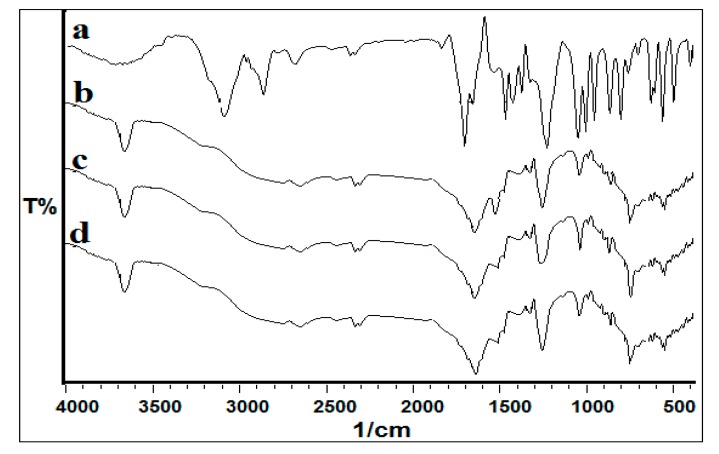
FTIR analysis of acephate (**a**), and its metabolites after 30th day (**b**, **c** & **d**). Here **a**, **b,** and **c** represent the FTIR spectrum of metabolites with respect to *P. aeruginosa, P. putida,* and *P. azotoformans* strains.

**Figure 4:**
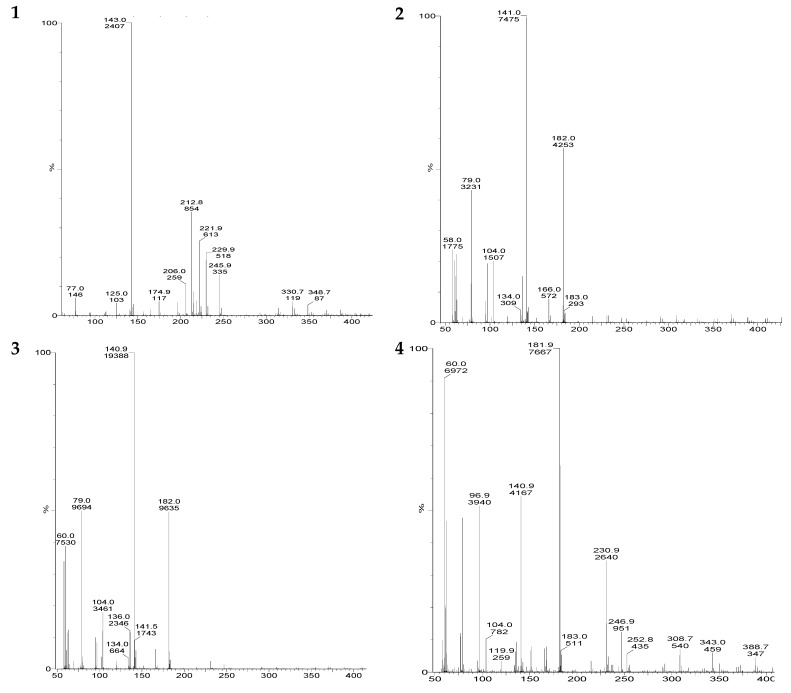
LC-MS(Liquid Chromatography–Mass Spectrometry) chromatogram (after 30th day) of (**1**) acephate + strain, (**2**) acephate + strain + Cu(II), (**3**) acephate + strain + Fe(III), and (**4**) acephate + strain + humic acid (here strain is *P. aeruginosa*).

**Figure 5:**
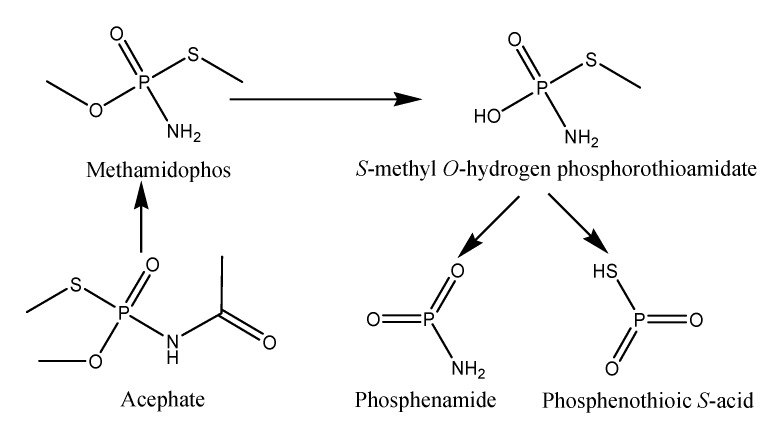
Most probable mechanistic pathway of decomposition of acephate in M9 medium.

The mass spectrometric method (ESI-MS) is considered as the best technique to determine polar and multi-matrix pesticides in their active and decomposed form [14,15]. The results indicate that all three strains were able to decompose acephate in M9 medium (Table 2 and Figure 2). The acephate removal rates were similar to the results of cell growth obtained with UV—visible spectroscopy. Removal of acephate increased with an increase in the incubation period. Figure 3 shows that, under different conditions, the maximum removal of acephate was shown for the *P. aeruginosa* followed by *P. putida* and *P. azotoformans*. Without application of humic acid and metal ions, the observed removal ranged from 69 to 77%, whereas 64 to 68%, 60 to 66%, and 69 to 74% removal was observed with the application of Fe(III), Cu(II), and humic acid, respectively. The overall order of acephate removal under various conditions was *P. aeruginosa* > *P. putida* > *P. azotoformans*. In the present study, the decomposition pathway in the absence and presence of humic acid and metal ions was similar. The observed major metabolites were methamidophos (m/z 143.11), S-methyl O-hydrogen phosphorothioamidate (m/z 125.0), phosphenothioic S-acid (m/z 95.0), and Phosphenamide (m/z 77.0) (Figure 4). The most probable mechanistic pathway of decomposition of acephate in M9 medium with and without application of humic acid and metal ions is depicted in Figure 5.

Mass spectrophotometry analysis was supported by FTIR analysis, where a change in functional groups was checked at regular time intervals, i.e., 3rd, 7th, 14th and 30th day. The FTIR spectrum of pure acephate exhibited peaks at 3115 cm^−1^ (N-H stretching), 1224 cm^−1^ (p=O stretching), 710 cm^−1^ (C-S stretching), 2829 cm^−1^ (C-H stretching of alkanes), and 1699 cm^−1^ (CO of -CO-NH- stretching) (Figure 3) (7,9,10). The observed spectra of FTIR on the 30th day of decomposed metabolites showed stretching of the hydroxyl group peaks at 1250 cm^−1^ for O-H and stretching of the amine group at 1646 cm^−1^ for NH_2_ (Figure 3). The changes observed in peak pattern and shifting of functional groups confirmed the mineralization of acephate into different metabolites. The data of FTIR analysis strongly supported the formation of metabolites as described in the mass analysis.

### 3.4. Biodegradation Kinetics

Pseudo-first-order kinetics was applied to calculate the half-life period of removal of acephate under various conditions (Table 2). The overall order of removal of acephate was Inoculum + acephate > Inoculum + acephate + humic acid > Inoculum + acephate + Cu(II) > Inoculum + acephate + Fe(III). The observed half-life (t_1/2_) values were 14.33–16.72 d^−1^ (strain(s) + acephate), 18.81–21.50 d^−1^ (strain(s) + acephate + Cu(II)), 20.06–23.15 d^−1^ (strain(s) + acephate + Fe(II)), and 15.05–17.70 d^−1^ (strains + acephate + HA). Based upon the results shown in Table 2, the biodegradation efficiency of the three bacterial strains can be ordered as *P. aeruginosa* > *P. putida* > *P. azotoformans*. The observed results were significantly different at *p* < 0.05. Non-significant comparisons were noticed for *P. putida* and *P. azotoformans* under the stress of Fe (III) and humic acid (represented by a* in Table 2). Hence, the rate of acephate removal of bacterial strains *P. putida* and *P. azotoformans* was the same under the stress of Fe(III) and humic acid, but lesser than that of *P. aeruginosa*.

## 4. Discussion

Microbe-based removal of pesticides and other chemicals is considered as the best and most cost-effective technique. The microorganisms not only decompose the pesticides but also utilize them as a source of nitrogen and carbon. However, the decomposition of pesticides in the soil has some disadvantages, which include a complex matrix of soil and the potential alteration of the decomposition rate as well the mechanisms of decomposition of pesticides. In the present study, the aim was to check the removal of acephate under different complex conditions using three bacterial strains. The isolated bacterial strains were identified by biochemical tests according to well-described methods [7,9,10,12]. The bacterial isolates were reconfirmed by molecular biotechnology. There were various reports regarding biodegradation of OPs, including methyl parathion, ethoprophos, and chlorpyrifos [16,17]. Only a few reports are available on the bacterial-promoted biodegradation of acephate [7,9,10,12]. Previous studies reported that the removal of acephate takes place with the formation of metabolites like methamidophos (major metabolite), including O-methyl phosphoramidate, O,O-dimethyl phosphoramidate, and O,S-dimethyl phosphorothioate (DMPT) [9,10]. Recently, an acephate-degrading strain, *Pseudomonas* sp. Ind01, was isolated, capable to utilize acephate to methamidophos and acetic acid [10]. In another study, *Pseudomonas aeruginosa* strain Is-6 was isolated, which was capable of complete mineralization of acephate to methamidophos and acetic acid, and hydrolyzing methamidophos and other tested organophosphorus compounds such as methamidophos, dimethoate, parathion, methyl parathion, chlorpyrifos, and malathion [9]. The two above-mentioned studies revealed that acephate decomposes to form toxic metabolite methamidophos [9,10].

In the present study, three strains were able to decompose acephate in M9 medium under the stress of humic acid and metal ions. The reduction rate of acephate was time- and concentration-dependent. Acephate decomposition was more than 68% (i.e., 68–78%) without the application of humic acid or metal ions. The rate of decomposition was significantly (at *p* < 0.05) dropped with the application of humic acid, ranging from 69 to 74%. Initially, the rate of decomposition significantly varied, but after the 7th day, the % age decompositions were enhanced in the presence of humic acid. These results agree with those of Shehata et al. (2014), who also reported that humic acid could neutralize the adverse effects of a pesticide by inhibiting its active sites [18]. The initial rate was slowed down by the association dissociation mechanism between acephate and humic acid, which leads to the formation of aggregates through H-bonding and Van der Waals bonding [19,20]. The aggregate formations lead to the reduction in toxicity of acephate. Consequently, the microbes under study decomposed acephate at a good rate after 7 days, once the aggregate formations took place.

Metal ions can play a vital role in the soil surface and water matrix. Humic acid, along with copper and iron, is the most significant component in the environmental system and can play a significant role to remove xenobiotic compounds under natural conditions. In the present study, acephate removal rates were affected in the presence of Cu(II) and Fe(III). The observed rates ranged from 64 to 68% and 60 to 65% in the presence of Cu(II) and Fe(III), respectively. Overall, with the application of Cu(II) and Fe(III), 10 to 15% decrease was noticed on the decomposition rate of the three bacterial strains. These interactions were logical and expected as per the HASB (Hard and Soft Acids and Bases) principle. The better removal rate of the three bacterial strains under the stress of humic acid and metal ions was the major finding of the current study, and it may be applied under natural conditions. Previously, the degradation of acephate by microbial populations was reported by various authors [7,9,10], but the decomposition kinetics of organophosphates using acephate was never reported. The reason behind the slow bio-decomposition of acephate was the complex formation behavior of Fe(III) with acephate through O and N atoms of acephate. Here, Fe(III) may form a six-membered ring through the O of P=O and C=O, which is kinetically as well as thermodynamically stable [17].The mass study revealed that the pathway of decomposition of acephate was similar to that of decomposition without the application of metal ions. Here, the slow removal rate of acephate was attributed to the toxicity of metal ions as well as the complex-formation ability of acephate [7]. It is well documented that acephate can form stable complexes with metal ions, including iron and copper [7].

The decomposition rate of xenobiotic compounds depends upon factors like nature of pollutants (i.e., hydrophobic or hydrophilic), soil composition (metal ions, humic substances), types of microbes (bacteria or fungi), and environmental conditions (pH, temperature, moisture, humidity) [19,20]. Calculations of decomposition rates by applying various mathematical tools and kinetic models are considered as a significant part of research and development. Chai et al. (2010) applied the first-order kinetic model to determine the mineralization and degradation kinetics of acephate in humid tropic soils [13]. They found that the sorption ability of acephate was low, that acephate could easily leach out into the aquatic environment, and that it decamped faster in air-dried soils (i.e., t_1/2_ 9–15 days); the decomposition rate significantly decreased in wet soils (i.e., t_1/2_ 32–77 days) and sterilized soils (i.e., t_1/2_ 53–116 days). In the current study, the decomposition rates of acephate were analyzed under wet conditions, and removal rates (i.e., t_1/2_ 11–23 days) were far better than in the previous study of Chai et al. (2010) [13].

The photo-catalytic based decomposition analysis of OPs has been reported using the kinetic models, where two OPs (chlorpyrifos and phoxim) were decomposed under Ag(I) stress, using first-order exponential decay kinetics [21]. In the present study, the kinetic model revealed that the decomposition of acephate was between 14 to 24 days. As mentioned above, the application of humic acid and metal ions inhibited the decomposition rate. The smallest adverse effect was noticed in the case of humic acid, followed by copper and iron metal ions.

## 5. Conclusions

The results of the present study indicate the utility of isolated bacterial strains to decompose acephate under the influence of metal ions and humic acid. In light of this and previous results, the use of isolated bacterial strains may allow cleaning of water and soils through the biodegradation of pesticides, including acephate. In the present report, results appear more effective due to its natural and practical approach, i.e., the bioremediation of OPs-contaminated soils under the stress of metal ions and humic acid.

## Figures and Tables

**Table 1:** UV—visible study (at 600 nm) for cell growth (%) under the influence of acephate/humic acid/metal ions by three bacterial strains in M9 medium.

	Time Period (days)			
Experimental Conditions	3	7	14	30
Cell Growth (%) with *Pseudomonas aeruginosa*				
Inoculum + acephate	14 ± 0.85 ^a,b^	25 ± 2.44 ^a,b*^	42 ± 3.44 ^a,b*^	77 ± 4.55 ^a,b^
Inoculum + acephate + Cu(II)	10 ± 0.71 ^a,b*^	22 ± 1.05 ^a,b^	43 ± 3.21 ^a,b*^	67 ± 3.72 ^a,b*^
Inoculum + acephate + Fe(III)	10 ± 0.79 ^a*,b*^	26 ± 2.85 ^a,b*^	39 ± 2.97 ^a*,b^	66 ± 3.44 ^a,b*^
Inoculum + acephate + HA	8 ± 0.57 ^a*,b^	28 ± 2.45 ^a*,b^	47 ± 3.01 ^a,b^	74 ± 4.22 ^a,b^
Cell Growth (%) with *Pseudomonas putida*				
Inoculum + acephate	11 ± 0.65 ^a,b*^	27 ± 1.58 ^a,b*^	54 ± 2.95 ^a,b^	74 ± 4.55 ^a,b^
Inoculum + acephate + Cu(II)	9 ± 0.25 ^a*,b^	24 ± 1.58 ^a,b*^	39 ± 2.33 ^a,b^	64 ± 3.09 ^a*,b^
Inoculum + acephate + Fe(III)	7 ± 0.38 ^a,b^	25 ± 1.11 ^a,b*^	33 ± 2.08 ^a,b^	60 ± 4.27 ^a*,b^
Inoculum + acephate + HA	11 ± 0.61 ^a,b*^	26 ± 1.44 ^a,b*^	44 ± 2.81 ^a,b^	71 ± 4.92 ^a,b^
Cell Growth (%) with *Pseudomonas azotoformans*				
Inoculum + acephate	12 ± 0.66 ^a,b^	22 ± 1.25 ^a,b*^	41 ± 2.58 ^a,b^	69 ± 4.11 ^a,b*^
Inoculum + acephate + Cu(II)	9 ± 0.40 ^a*,b^	18 ± 1.03 ^a,b^	37 ± 2.08 ^a,b^	64 ± 3.55 ^a*,b^
Inoculum + acephate + Fe(III)	10 ± 0.53 ^a*,b^	23 ± 1.44 ^a,b*^	39 ± 2.35 ^a*,b^	61 ± 3.77 ^a*,b^
Inoculum + acephate + HA	8 ± 0.33 ^a*,b^	28 ± 1.42 ^a*,b^	45 ± 2.48 ^a,b^	69 ± 4.02 ^a,b*^

a = results significantly differed (at *p* < 0.05) for three strains in the same experimental conditions. a* = results did not differ significantly (at *p* < 0.05) for two or more than two strains in the same experimental conditions. b = results significantly differed (at *p* < 0.05) for each strain in four different experimental conditions. b* = results did not differ significantly (at *p* < 0.05) for each strain in different four experimental conditions.

**Table 2:** Kinetics of biodegradation of acephate by three bacterial strains in M9 medium.

Experimental Conditions	K	t_1/2_ (days)	Equation of line	r^2^
*Pseudomonas aeruginosa*				
Inoculum + acephate	0.021	14.33 ^a,b^	y = −0.021x + 2.024	0.98
Inoculum + acephate + Cu(II)	0.016	18.81 ^a,b^	y = −0.016x + 2.002	0.94
Inoculum + acephate + Fe(III)	0.015	20.06 ^a,b^	y = −0.015x + 1.991	0.96
Inoculum + acephate + HA	0.020	15.05 ^a,b^	y = −0.02x + 2.009	0.97
*Pseudomonas putida*				
Inoculum + acephate	0.019	15.84 ^a,b^	y = −0.019x + 2.009	0.97
Inoculum + acephate + Cu(II)	0.014	21.50 ^a,b^	y = −0.014x + 1.993	0.98
Inoculum + acephate + Fe(III)	0.013	23.15 ^a*,b^	y = −0.013x + 1.992	0.96
Inoculum + acephate + HA	0.017	17.70 ^a*,b^	y = −0.017x + 1.999	0.95
*Pseudomonas azotoformans*				
Inoculum + acephate	0.018	16.72 ^a,b^	y = −0.018x + 2.012	0.97
Inoculum + acephate + Cu(II)	0.015	20.06 ^a,b^	y = −0.015x + 2.011	0.98
Inoculum + acephate + Fe(III)	0.013	23.15 ^a*,b^	y = −0.013x + 1.983	0.96
Inoculum + acephate + HA	0.017	17.70 ^a*,b^	y = −0.017x + 1.992	0.97

a = results significantly differed (at *p* < 0.05) for three strains in the same experimental conditions. a* = results did not differ significantly (at *p* < 0.05) for two or more than two strains in the same experimental conditions. b = results significantly differed (at *p* < 0.05) for each strain in four different experimental conditions.

## Supplementary Materials

The following are available online at https://www.mdpi.com/2218-273X/10/3/433/s1, Figure S1: Bacteria isolated from acephate contaminated soils, Table S2: Biochemical characterization of isolated bacterial strains.
