# Supplementary material for: Kinetic Study of the Biodegradation of Acephate by Indigenous Soil Bacterial Isolates in the Presence of Humic Acid and Metal Ions
Source: Biomolecules. 2020 Mar 11;10(3):433. doi: 10.3390/biom10030433 (PMC7175145; doi:10.3390/biom10030433)
Supplement: Supplementary file 1 [file biomolecules-10-00433-s001.pdf]

Supporting Data

Biodegradation of Acephate using Three Bacterial Strains and Effect of Humic Acid and Metal Ions

Figure - S1: Isolation of bacteria from acephate contaminated soils.

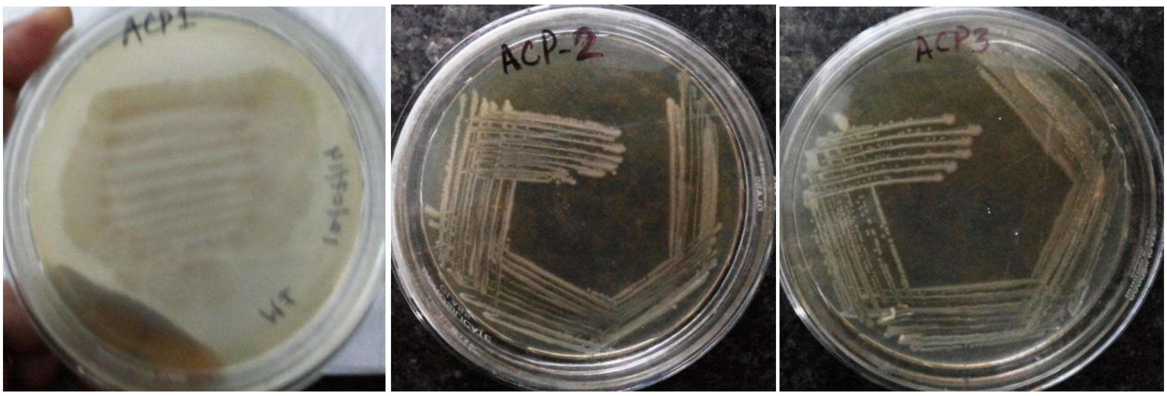

Table – S2: Biochemical characterization of isolated bacterial strains.

S No.	Biochemical tests	*P azotoformans*	*Paeruginosa*	*P putida*
1.	Carbohydrate	Positive	Positive	Positive
2.	Citrate test	Positive	Positive	Positive
3.	Indole test	Positive	Positive	Positive
4.	MR test	Negative	Positive	Positive
5.	VP test	Positive	Negative	Negative
6.	Nitrate test	Positive	Positive	Positive
7.	Starch hydrolysis	Positive	Positive	Positive
8.	Tryptophan test	Negative	Negative	Negative
9.	Motility test	Negative	Negative	Negative
10.	Glycerol test	Positive	Positive	Positive
